# Mortality Analysis of Patients with COVID-19 in Mexico Based on Risk Factors Applying Machine Learning Techniques

**DOI:** 10.3390/diagnostics12061396

**Published:** 2022-06-05

**Authors:** Aldonso Becerra-Sánchez, Armando Rodarte-Rodríguez, Nivia I. Escalante-García, José E. Olvera-González, José I. De la Rosa-Vargas, Gustavo Zepeda-Valles, Emmanuel de J. Velásquez-Martínez

**Affiliations:** 1 Unidad Académica de Ingenieía Eléctrica, Universidad Autónoma de Zacatecas, Zacatecas 98000, Mexico; armandorodarte19@gmail.com (A.R.-R.); ismaelrv@ieee.org (J.I.D.l.R.-V.); gzepeda@uaz.edu.mx (G.Z.-V.); iemmanuelvm@gmail.com (E.d.J.V.-M.); 2 Laboratorio de Iluminación Artificial, Tecnológico Nacional de México Campus Pabellón de Arteaga, Aguascalientes 20670, Mexico; aivineg82@gmail.com (N.I.E.-G.); e.olvera.Itp@gmail.com (J.E.O.-G.)

**Keywords:** COVID-19, mortality analysis, risk factors, machine learning

## Abstract

The new pandemic caused by the COVID-19 virus has generated an overload in the quality of medical care in clinical centers around the world. Causes that originate this fact include lack of medical personnel, infrastructure, medicines, among others. The rapid and exponential increase in the number of patients infected by COVID-19 has required an efficient and speedy prediction of possible infections and their consequences with the purpose of reducing the health care quality overload. Therefore, intelligent models are developed and employed to support medical personnel, allowing them to give a more effective diagnosis about the health status of patients infected by COVID-19. This paper aims to propose an alternative algorithmic analysis for predicting the health status of patients infected with COVID-19 in Mexico. Different prediction models such as KNN, logistic regression, random forests, ANN and majority vote were evaluated and compared. The models use risk factors as variables to predict the mortality of patients from COVID-19. The most successful scheme is the proposed ANN-based model, which obtained an accuracy of 90% and an F1 score of 89.64%. Data analysis reveals that pneumonia, advanced age and intubation requirement are the risk factors with the greatest influence on death caused by virus in Mexico.

## 1. Introduction

Internationally, health sector constantly has a high demand for clinical care; there are many reasons that influence the overload in the quality of medical care and also cause a saturation of patients. The factors that generate a clinical saturation are diminished personnel, insufficient or well-qualified medical personnel, deficient infrastructure, ventilators, delay in medical diagnosis, hospitalization time, lack of medicines, among others [1,2,3,4,5]. Nowadays, an example of saturation of the health sector worldwide is the Coronavirus pandemic (COVID-19), which has caused an overflow in the quality and capacity of medical care in many of the clinical centers. This situation is also caused due to the high number of infected patients with COVID-19, until 17 April 2022, over 500 million confirmed cases and over 6 million deaths have been reported globally [6,7]. These numbers continue to increase and cause problems in clinical demand. Another problem consists in the difficulty to give an accurate diagnosis in a short time, without forgetting that this diagnosis can be somewhat subjective. There are several factors that influence to achieve a correct medical assessment, such as the professional capacity and specialty of the staff, degree of studies, own judgment, stress, assessment of the patient, fatigue of personnel, among others [8,9,10,11].

Coronavirus pandemic has led to the demand for the application of various modern techniques and technologies such as artificial intelligence (AI) tools, which has multiple applications in fields of science such as medicine, meteorology, astronomical exploration, including applications in financial systems, among others [12,13,14,15]. In this sense, IA makes it possible to design schemes in order to speed up medical diagnoses, reduce the saturation of clinical centers, increase the quality of medical care, obtain better medical assessment and solve various other problems [12,13,14,15,16,17,18,19]. Thus, novel models need to be developed to support medical personnel, allowing them to be an aid in diagnosing more efficiently health status of a COVID-19 infected patient.

Accordingly, IA models can use risk factors associated with the death as guideline and predict the probabilities that a patient will die or recover (health status diagnosis) from this new Coronavirus. Risk probability results are helpful in making better quality clinical decisions in a limited time environment and with limited resources, obtaining more detailed and accurate clinical diagnoses. Consequently, health risk factors plays an important role, increasing the chances of developing a serious COVID-19 infection (suffering complications) and causing death, or at least increasing its chances. In addition, these factors can originate complications, and therefore be taken to an intensive care unit (ICU) [20,21,22,23,24,25,26,27,28,29,30,31,32,33,34]. A logistic study with several variables indicated that factors such as chills, body temperature, initial chest X-ray findings and diabetes were valued as elements that cause the aggravation of the health status of patients with COVID-19. This is probably because some of these factors cause hyperglycemic conditions that generate immune dysfunction, as well as impaired neutrophil function, humoral immunity and antioxidant system function [35]. In other previous studies, age was reported to be the most important predictor of death in COVID-19 patients. In this sense, infected older people often suffer severe complications, mainly due to age-dependent functional defects in immune cells that lead to poor suppression of viral replication [35,36].

Another risk factor to consider in modeling these IA schemes is cardiac injury, where the mortality rate is higher among patients with this disease in relation to those who do not have it. COVID-19 patients with cardiac injury experienced greater severity, evidenced by abnormal radiographic and laboratory results, among which are: NT-proBNP, elevated levels of C-reactive protein and creatinine levels, as well as more multiple mottling and opacity in ground glass; where a higher proportion required non-invasive or invasive ventilation [37]. Severe pneumonia was also independently associated with admission to intensive care unit, mechanical ventilation, or death in the competitive-risk multivariate model due to the cause of more severe respiratory problems [38]. Besides, regarding recent information, obesity is a factor that originates the aggravation of the disease, causing people to require hospitalization and ventilation mechanisms. In this way, obesity induces alterations in the microbiota and physiological and immune responses, which are related to deficient viral responses [39].

Taking into account this information, there are several types of AI applications based on various variables in the COVID-19 pandemic, in addition to abundant and diverse works for each type of application [16,17]. For example, the early detection and diagnosis of infection, which aims to analyze irregular symptoms and other “warning flags”, and thus help make decisions faster. Another application lies in reducing the workload of healthcare staff, assisting in the early diagnosis of the virus and providing medication in a timely manner using some kind of data analysis process [40]. Also this supplies opportunities to improve abilities of medical workers with respect to the new challenges of the disease [12,16,18].

Regarding the projection of COVID-19 cases and mortality, the aim is to foretell the number of sick and unrecovered people in a specific country; furthermore, it can help recognize which type of people is vulnerable. Likewise, there is a great variety of studies of this type despite the short time that has elapsed since the pandemic began [16,41]. We can find research around the world about this topic employing IA techniques such as random forest models [17,42,43,44,45], deep learning [46,47,48,49,50,51,52,53], decision trees [43,54], support vector machine (SVM) [49,55] and logistic regression procedures [49,56]; which are intended to predict the health status (mortality risk or disease severity) of a COVID-19 infected patient employing factors such as the patients age, weight, gender, physiological conditions, demographic data, travel data, computed tomography, vital signs, symptoms, smoking history, radiological features, clinical features, genetic variants, platelets, laboratory test, D-dimer test, chronic comorbidities and general health information. Meantime, other studies [57] create models using data analysis techniques with the aim of predicting the need of oxygen therapy in a timely manner in COVID-19 patients; which employed variables like shortness of breath, cough, age and fever. We can also find related works intended to design CNN-based models with the purpose of detecting positive cases of COVID-19 using chest X-ray images [58].

This research defines AI models taking into account risk factors to predict the health status (chances of recovering or not recovering) of patients infected by COVID-19 in Mexico. Thus, this proposal can support medical workers to evaluate patients more quickly and accurately due to overload in hospitals; likewise, they can also provide better quality care based on priorities for risk of severity or death. To accomplish the task, various machine learning and learning mathematical models are implemented and compared for prediction and classification, such as logistic regression, KNN, decision trees, neural networks and random forests. To the best of our knowledge, there is no AI-based model in the state-of-the-art review that considers the same predictive variables, thus making it an alternative support approach. In this sense, the implementation of the algorithms is carried out by performing the search for squares and cross validation to fit the models, which is applied on a open dataset provided by the Secretary of Health of Mexico. This corpus of data collects information on different risk factor variables (16 risk factors and other variables), which are associated with death in patients with COVID-19. This dataset contains the accumulated information (epidemiological study) of all suspected and confirmed cases of the viral disease COVID-19 in Mexico; furthermore, these cases are found within different units of the Mexican Health Sector [59]. With these proposal, ANN was the suggested model to predict the health status (risk probability of dying) that patients with COVID-19 have, obtaining an accuracy of 90.02%, a precision of 92.60% and F1 score of 89.64%; while for the recall a 86.87% was obtained. The suggested results do not present high variance problems, but high bias problems. The results showed that the variables (risk factors) with the greatest importance or influence on death caused by COVID-19 are intubation requirement with 47%, pneumonia with 34% and age with 14%.

Due to the similar objective of other existing works, it is worth mentioning that this study is under other conditions and has been carried out in different country or region. Each site has its own circumstances and may differ from place to place (causing a different pattern in the statistics) in terms of the quality of medical care (hospital infrastructure, quality of care, professional training). In addition, other factors are involved, such as population with obesity, eating habits, average age of population, quality of health (diseases suffered by age), number of infected by age, general habits of population like smoking, among others. For this reason, the main contributions of this paper are emphasized:

- A mortality predictor model based on risk factors for COVID-19 is presented, which is trained with data from the open dataset “COVID-19 databases in Mexico” provided by the Secretary of Health of Mexico. The proposed scheme uses different data and predictor variables regarding the state-of-the-art, while being applied in different region and circumstances.
- An analysis of the relationship of variables used and their interpretation is carried out.
- This work proposes an AI model to identify risk factors with the greatest influence on death from COVID-19 in Mexico.
- This work applies the grid search technique in a nested way with cross validation to improve performance and evaluate the proposed algorithms.
- This analysis will allow researchers to continue working on the development of new models to predict the health status of patients, and will allow to identify new variables with influence on death caused by COVID-19, especially in Mexico.

The rest of the paper is organized as follows. Section 2 presents the proposal of this work, which describes the statistical analysis carried out and the basic idea behind the proposed models. The evaluation and results of the models as well as their comparisons are shown in Section 3. A discussion of the results and their corresponding comparison is described in Section 4. Finally, Section 5 concludes the paper with a brief analysis and future work.

## 2. Prediction of Health Status in Patients Infected by SARS-CoV-2

### 2.1. Proposed Framework

After selecting the best subset of features, several machine learning algorithms were implemented to build a predictive model and perform a comparison. In this research, we use different algorithms including majority vote, artificial neural networks, random forests, decision trees, logistic regression, and K-nearest neighbor (KNN).

**Figure 1:**
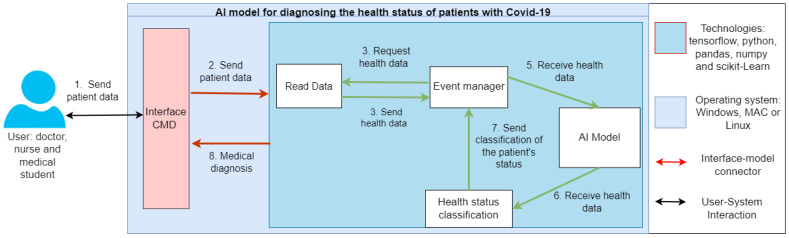
General proposal diagram for diagnosis of COVID-19 patients health status.

The general architecture of the proposed model is based on Figure 1, where user provides clinical information of the patient in turn, and later this input is supplied to the diagnostic system of patients health status. Within this model, the data reading process is generated, which interacts with the event handler mechanism. The event handler then channels the flow to the health state prediction algorithm; later flow is sent to the classification model so that it can be channeled back to the event handler. Previous information generated and accumulated is given in the form of a prediction (probabilities and classification) and as output to end user. All this process is reflected through a computer program to generate/store the respective information, which will be sent as output to user in support of the requested diagnosis. In order to meet the expected objectives, the computer must have different required technologies installed showed in the diagram.

**Figure 2:**
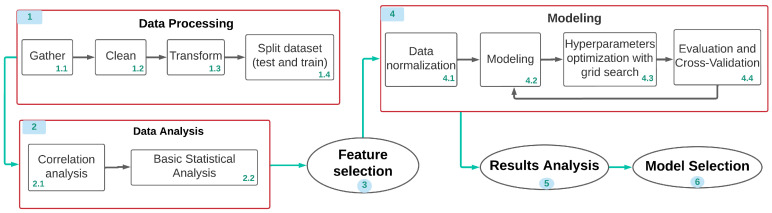
Overview diagram of data processing, analysis and modeling for the proposal.

Figure 2 sequentially describes the data processing, analysis and modeling procedures. The first step is to manipulate the data, in this phase the data is obtained and cleaned (eliminating corrupted or unnecessary records), later this data is transformed to make proper use of it. The second phase is to analyze the data to summarize the properties of each variable, find relationships between variables and understand their behavior. Later (phase 3), an intelligent algorithm is applied, within this modeling phase the data is normalized, hyperparameters are optimized and the model is evaluated. This is repeated for all models and their different configurations (k models). After obtaining the first model (random forests), this is used to select the most important features (phase 4); this procedure is only done once. Once all the models have been obtained, we proceed to phase 5, where the results are optimized, compared and analyzed. Finally, from the analysis of the results, the most optimal model is selected and proposed as a solution (phase 6).

**Figure 3:**
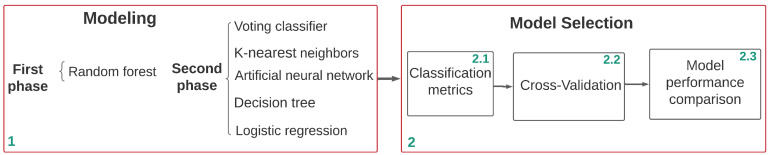
Diagram of modeling for the proposal.

Figure 3 details the selection process for the best model. Once the different algorithms proposed have been modeled (phase 1), the results of these are analyzed. As a final stage (phase 2) to select the best model, cross-validation is implemented to measure the performance of different models in unseen data (detect if there is a memorization of the data). Then, metrics such as confusion matrix, precision, accuracy, recall and F1 score are used to individually evaluate these models. Finally, the different algorithms are compared to select the model that presents the least memorization of data (overlearning) and the highest precision (reliability) and recall (ability to differentiate).

### 2.2. Mobile App COVID-19

**Figure 4:**
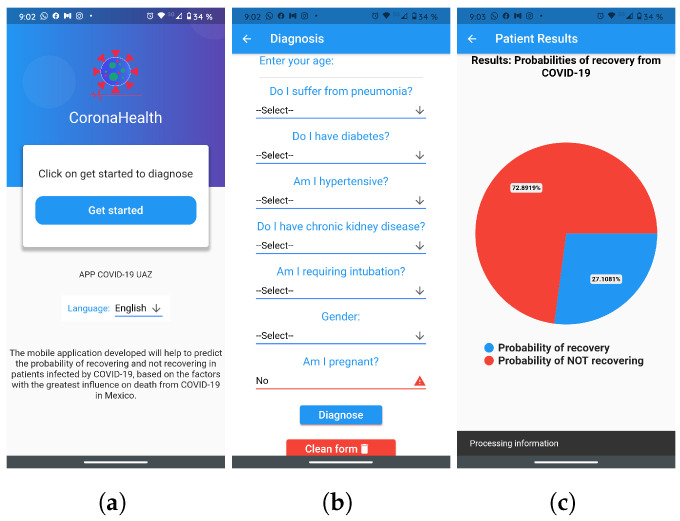
Mobile app CoronaHealth (Interfaces). (**a**) Home; (**b**) Form; (**c**) Results.

In order to address the prediction of mortality from COVID-19 based on risk factors, the design and implementation of a mobile app has been proposed as a guideline (whose name is CoronaHealth, see Figure 4), which will serve as support for the diagnosis of patients at risk of dying due to this disease. This app is made up of the best compared scheme (neural network) as the proposed predictive model. The application collects only information on the patient’s risk factors in the form of a short questionnaire. Subsequently, the neural network model makes the diagnosis based on the risk factors; and finally, the results are shown in a graph, where it indicates the patient’s chances of dying or recovering.

### 2.3. Data Overview

For this study, the open dataset “COVID-19 databases in Mexico” was used, which was obtained from the official web page of the Secretary of Health. This collects information on different risk factor variables, which are associated with death in patients with COVID-19 [7,59]. However, this dataset has been previously processed to meet the needs of this research. From the extended initial dataset, different subsets processed in this work were formed. Table 3 presents data features for the subset used, where the possible values of variables are Yes (1), No (0), Ignored (3). In the first dataset version, patients with some unknown risk factor were excluded. The second dataset version does consider the patients with some unknown risk factors; the different causes, for which the suffering of some risk factor was unknown, were grouped in a new categorical value (ignored).

It is important to note that several cuts were made from the dataset (see Table 1); where the total classes to predict is always one, the “death” variable (recovered or unrecovered patients). Each one with different (quantity) data, and some of them (recent versions) contemplate the hypothesis that some patients were vaccinated (whose information is unknown). We first present the analysis from the initial cut made until 15 December 2020; where this cut provides a guideline for the subsequent cuts. Accordingly, in the results part, the analysis of other cuts made until now (April 2022) are presented.

### 2.4. Data Processing

These different datasets or data cuts have been previously processed by us to meet the needs of this research. In this phase, data was adapted for the different models that will be applied to them, based on the raw data; e.g., merged, sort data and attributes, besides, incomplete records are also removed, including the mapping of boolean and/or categorical values from string to numeric. The data corpus used was divided (each one) into training and testing. The training set contains 75% of the cases in each category (deceased or recovered persons). For example in first cut, training set has a total of 179,098 COVID-19 cases, of which 87,323 patients died and 91,775 survived [7]. While the testing set contains the remaining 25%, i.e., it contains 59,700 cases, of which 29,164 patients died and 30,536 recovered. A similar number was maintained between discharged and deceased patients in both data subsets, avoiding class imbalance as much as possible. However, the number of elements was filtered to have a more homogeneous number of cases between deceased and surviving patients in the two sets formed and used.

### 2.5. Data Analysis

A statistical analysis was carried out to understand the behavior of the data and the relationship between them.

#### 2.5.1. Correlation

The correlation between the risk factors in the dataset (see Table 2) provides salient information on the features and the degree of influence they have on the target value (the variable “death”), according to [60,61]. Thus, there is a strong positive correlation between the variables pneumonia, ICU and intubated with the variable death, and a strong negative correlation between the variable age and death. Also, it is observed that these same variables are highly correlated with each other. Based on this analysis, it can be deduced that COVID-19 patients with pneumonia and advanced age are more likely to die, be intubated or enter the ICU. Furthermore, this analysis indicates that people with diabetes, advanced age, hypertension and pneumonia are more prone to complications and to be taken to the ICU or to be intubated.

Table 2 shows a good correlation of the death variable with the intubated, pneumonia, age, diabetes, hypertension and ICU variables, with correlations of 0.692, 0.675, −0.565, 0.289, 0.311 and 0.651, respectively. Also, there is a low correlation between the gender variable and the death variable with 0.112. In addition, there is a good correlation of the age variable with respect to the variables intubated, pneumonia, diabetes, hypertension and ICU, with correlations of −0.490, −0.470, −0.291, −0.383 and −0.445, correspondingly. Similarly, there is a good correlation of the ICU variable with the variables intubated, pneumonia, diabetes and hypertension, with correlations of 0.927, 0.885, 0.248 and 0.249, respectively. The variable diabetes obtains a low correlation with the variables intubated with 0.271 and pneumonia with 0.262. Finally, there is a slight correlation of the hypertension variable with respect to the variables intubated, pneumonia and diabetes, with correlations of 0.277, 0.264 and 0.404, correspondingly.

If any categorical value of the variables is removed, so this would cause loss of data information and continuity value in the variables, therefore, it results in the loss of a correlation and existence of arbitrary limits. Also, having a loss of information eliminates possible false relationships possibly due to: (a) two variables have a relationship that is not linear, so the relationship is not detected, (b) the data have outliers, (c) the data have two subgroups with little correlation within them, and (d) the values variability on the *Y* axis changes with the corresponding values on the *X* axis [60,61].

However, it can be concluded that there is a linear relationship between certain variables. Pneumonia and age tend to aggravate the disease, causing the patient to require intubation. The worsening of the disease, advanced age and suffering from pneumonia have a high linear relationship with the death (risk factors with a high influence on it). Finally, at an older age it can be seen that people tend to suffer from certain diseases with a high influence on death, such as hypertension, diabetes and pneumonia. By keeping the category “ignored”, important information related to the mortality of patients with COVID-19 is preserved. Correlation analysis has several solutions, while feature importance analysis can be used to check the degree of data relationship.

#### 2.5.2. Basic Analysis

**Figure 5:**
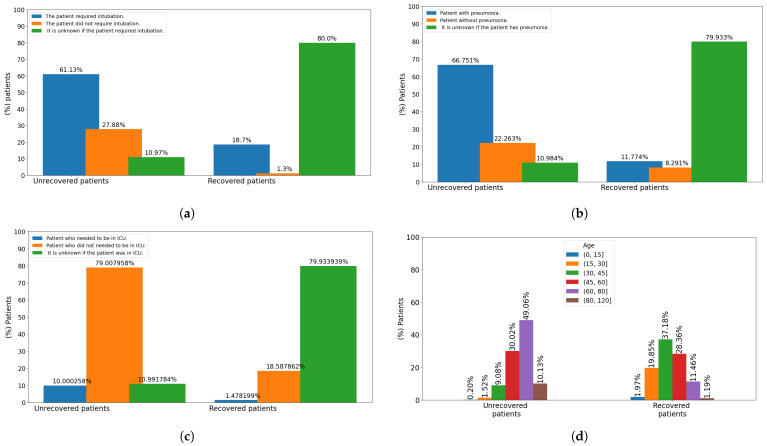
Basic dataset analysis. (**a**) Intubated patients; (**b**) Patients with pneumonia; (**c**) Patients in ICU; (**d**) Patients by age.

Most of the patients (95%) have an age between 34 and 70 years old; there is a maximum age of 119 and a minimum of 0 years for babies infected by COVID-19. In addition, most of the non-recovered patients are between 60 and 80 years old, while most of the recovered patients are between 30 and 45 years old (see Figure 5d). Moreover, 57.8% of the patients analyzed in this study are men, while 42.19% of the patients correspond to women. 36.5% of the deceased people have been female, while 52.3% of the recovered persons are men. Otherwise, 61.13% of the deceased persons required intubation, 10.97% of the unrecovered people are unknown if they required any intubation, whilst 27.88% of the deceased cases did not require any intubation (see Figure 5a). Besides, 80% of the recovered people do not know precisely if they needed to be intubated; however, 18.7% of the recovery cases required intubation. In this sense, 66.75% of the deaths presented pneumonia and only 11.77% of the recovered persons showed pneumonia problems (see Figure 5b). 38.5% of the patients in this study were diagnosed with pneumonia, 15.1% of the patients were negative for pneumonia and 46.3% of the cases is unknown for sure for different causes.

Relative frequency analysis of categorical data indicates that patients requiring intubation have pneumonia, and being male, tend to die and rarely recover. However, in most recovery cases, it is not known for sure whether the patient had pneumonia and required intubation. A possible limitation of this study is the existence of a possible bias in the collection of these data in clinical centers. In addition, 22.26% of the deceased patients do not present pneumonia and 10.98% of the death cases it is unknown if they presented pneumonia. Also, 8.29% of the recovered patients do not present pneumonia, while in 79.93% of the recovered patients the presence or absence of pneumonia is unknown (see Figure 5b). In this sense, 79% of the unrecovered patients did not need to be admitted to the ICU, unlike 10% who needed. On the other hand, 79.93% of the recovered people are not known if they needed to be admitted to the ICU, unlike unlike the 1.47% who did and the 18.58% who did not (see Figure 5c).

### 2.6. Feature Selection

A model based on random forests was trained in order to evaluate the importance of the dataset features. This technique was used to later choose the most important features for the formation of alternative prediction models. A random forest has been implemented in scikit-learn. Feature selection produces simpler models, because fewer parameters are required to be adjusted. It also allows the dimensionality reduction of the dataset, avoiding unnecessary variables and overfitting.

#### Random Forest Model for the Selection of Features and Identification of Their Importance

The higher the value, the more important the feature. The importance of a feature is calculated as the total (normalized) reduction of the criterion contributed by that feature. It is also known as the importance of Gini. This technique measures the importance of features such as the mean decrease in impurities generated from forest decision trees, without taking into account assumptions that have to do with whether or not linearly separable data exist [62,63,64]. With the purpose of dividing the nodes into more informative characteristics, a performance index is required, which will be optimized in the learning phase. The aim of this index is to maximize the information gain in each division of the tree, known as the Gini index (see Equation (1)) [65].

(1) $$IG\left(D_{p},f\right)=I\left(D_{p}\right)-\sum_{j=1}^{m}\frac{N_{j}}{N_{p}}I\left(D_{j}\right),$$

where *f* is the feature to define the division, $D_{p}$ is the dataset of the parent node, $D_{j}$ is the dataset of the child node, *I* corresponds to the measure of impurity, $N_{p}$ indicates the total number of samples from the parent node and $N_{j}$ is the total number of samples from the child node. As expressed in Equation (1), the information gain is the difference between the impurity of the parent node and the sum of the impurities of the child nodes. The less the impurity of the child nodes, the greater the information gain. For simplicity and less computational cost, binary trees are usually defined [65].

Table 3 describes the importance of dataset features, based on this analysis, the 8 most important features were selected to form different and alternative prediction models. All the models developed were tested with the 16 original features and the 8 most important characteristics. In this study, the 8 most important features considered are: sex, intubation, pneumonia, age, pregnancy, diabetes, hypertension and chronic kidney disease. These factors are the main causes or complications of death from Coronavirus in our dataset. Previous studies have reported that age is the most important predictor of death in COVID-19 patients [66]. On the contrary, in our study, age was not a main predictive factor of death, which obtained 14.89% of importance. This situation may be due to the fact that 90% of the patients included in our study are between 34 and 70 years old. Another factor in this circumstance is its high relationship with requiring-intubation variable. However, although the difference was not statistically significant, there is a tendency in older patients to progress to a severe stage of the disease, and thus it causes death compared to relatively younger patients.

Apart from age, requiring intubation is the main factor and with the highest mortality rate, with 47% of importance, since requiring intubation means an aggravation of the disease for various reasons. Requiring intubation was found as the most important variable because this variable represents an general abstraction and/or representation of variables associated with death from COVID-19 that were contemplated in this study; this due to its high correlation between them caused by the worsening of the illness. Age, pneumonia, diabetes and hypertension are highly correlated with the worsening of the symptoms or the disease (requiring intubation or entering the ICU), but especially the respiratory and lung problems that lead to the worsening of the disease. Pneumonia was also found as the second most significant risk factor with an importance of 34.5% and a high association with requiring intubation and/or entering the intensive care unit. This happends because it decreases the ability to breathe, as well as reduces its ability to allow the entry of oxygen and the expulsion of carbon dioxide, in addition, it can be caused by the new virus SARS-CoV-2 [66,67].

### 2.7. Modeling (Predictions Models)

Different machine learning algorithms were suggested and implemented comparing their performance. This was performed in order to compare the efficiency of their results, and thus be able to find the most optimal algorithm as a predictor of the mortality of people infected. Suggested models were implemented are KNN, decision trees, logistic regression, ANN and majority vote. With the aim of getting the best performance in each model, a complete results comparison of the 8 most optimized features as well as the total of 16 model features is performed. Project dependencies include the following packages and libraries: Numpy, Pandas, SciPy, Scikit-Learn, Tensorflow, and Matplotlib.

#### 2.7.1. Data Normalization

The normalization process is a crucial step, nevertheless, decision trees and random forests are one of the few algorithms that we should not worry about scaling features, since these are invariable to scaling. However, most algorithms work better if the features are on the same scale [68]. Standardization can sometimes be more practical in machine learning algorithms. The reason is because many linear algorithms initialize their weights very close to 0, which is why the columns take the form of a normal distribution and what makes weights learning more useful. Besides, this keeps useful information from outsiders and makes the algorithm less sensitive to them; for these reasons standardization was preferred.

#### 2.7.2. Random Forests for Feature Selection

A random forest scheme is composed of several decision trees, which partition a dataset into smaller parts, aggregating branches to the tree [69,70]. The outcome of this framework is a tree with leaf nodes and decision nodes. A decision node consists of several branches representing the value of each feature tested, while the leaf node contains the value of the result in the prospective condition of the patient (target value). A set of classifier decision trees provides a better model than a single decision tree in order to correctly predict a target value. Hence, the random forest averages the result obtained by the set of trees to generate the outcome [42]. Finally, the decision is the result of the sum of all the base models.

To form the model of random forests, the grid search was used with the purpose of adjusting its different parameters as best as possible. The precision was adjusted and the performance of the algorithm was evaluated to reduce overlearning and underfitting as much as possible. The optimal hyperparameters returned by the grid search algorithm are max_depth = 8, criterion = Gini, min_samples_split = 2, min_samples_leaf = 2 and n_estimators = 18, 12, 19 and 18 for the corresponding 4 data cuts. The decision trees that make up the algorithm have a depth equal to 8; furthermore, the Gini index at all leaf nodes in all trees is <0.5, indicating that the training dataset is unbalanced. Therefore, to optimize the performance of the model, it could be considered to reduce the depth of the trees and increase the number of estimators (decision trees) in the random forest to 100. This would avoid a high variation in the model and provide more accurate predictions. The application of the AdaBoost algorithm can also provide a corrective mechanism to improve the model after each prediction of the patient’s condition.

#### 2.7.3. Decision Tree

The most optimal hyperparameters to build the algorithm consists of max_depth = (between 8 and 10), criterion = Gini and min_samples_split = 2. Likewise, these parameters are obtained by grid search, such that the range of values to find the most optimal value of max_depth was the same, between 1 to 16. The depth of this tree is 9 nodes, which can be considered high in terms of the model complexity and could apply the pruning technique; thus somewhat limiting the capabilities of the model to obtain a machine learning model that is simpler and generalizes better. This current complexity of the model may become impractical in clinical practice for decision making.

**Figure 6:**
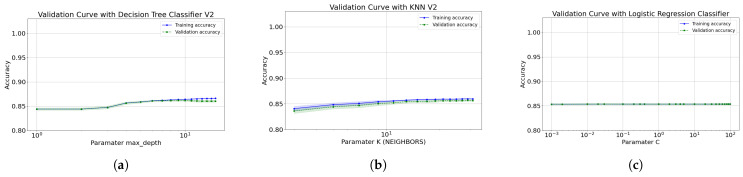
Validation curve for propounded models with 8 features, first data cut. (**a**) With decision tree; (**b**) With KNN; (**c**) With Logistic Regression.

**Figure 7:**
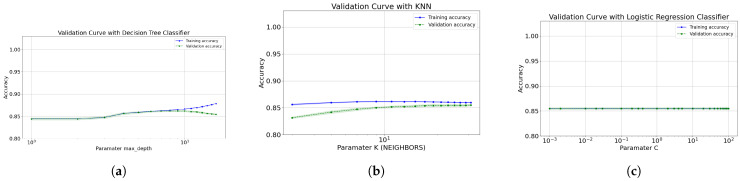
Validation curve for propounded models with 16 features, first data cut. (**a**) With decision tree; (**b**) With KNN; (**c**) With Logistic Regression.

Figure 6a and Figure 7a show the validation curve for the models on first data cut, which includes 16 and the 8 most important features. Also, with both features type this model tends to slightly over-adjust the data as the depth increases; where the decision tree reached an acceptable depth equal to 9. Furthermore, the Gini index at all leaf nodes in all trees is also <0.5, indicating that the training data set is also unbalanced. To optimize the performance of the model, it is proposed to apply the tree pruning technique; this avoids high variance in the model and provides accurate predictions. The same happens for configuration-1 of the decision tree and with the random forest algorithm already described.

#### 2.7.4. K-Nearest Neighbors (KNN)

Another proposed algorithm is K-nearest neighbors (KNN); this is a classification algorithm, as well as being supervised learning and vague. It is called vague not because of its apparent simplicity, but because it does not obtain any discriminative function from the training data, but instead memorizes the training dataset [71,72]. Principal component analysis (PCA) was applied using the sklearn library. Singular value decomposition, used by linear dimensionality reduction, projects data to a lower dimensional space. Thus, this input data is centered, but not scaled for each feature. The value of the parameter n_components = 0.95 indicates that the explained variance is between 95% and 99%, thus compressing the dataset *n* components smaller than the original [73].

Figure 6b and Figure 7b present the validation curve for the range of values defined for k closest neighbors. Essentially, the only interpretive bias that we cannot avoid in model is the choice of nearest neighbors number (k). Thus, the selection of k is a common limitation. In predictions where k is small, particularly k = 1, the variance per prediction is greater, creating a sub-learning. In noisy datasets, where the nearest neighbor is based on poor quality data, the unknown instance will lead to noisy predictions. However, in predictions using a large neighborhood, the predictions begin to be skewed (under-fits), as it happens. After k tests (from 1 to 31) and taking into account these limitations, we choose k = 31, balancing this value to be small enough to alleviate smoothing and large enough to reduce noise.

The election of data analysis algorithm is commonly addressed by performance on a particular context. Thus, k nearest neighbors algorithm was chosen due to its simplicity, as well as for allowing a minimal user input to yield results. “K” corresponds to the number of closest neighbors, which use the proximity in the parameter space. The most optimal hyperparameters to build the algorithm were n_neighbors = 31 and *p* = 2. These parameters are obtained by grid search. The range of values to find the most optimal value of closest neighbors was between 2 and 32, only considering the odd values. The parameter *p* indicates the type of metric for the distance, in this case the Euclidean distance was implemented to build the KNN algorithm. The best parameter returned by GridSearchCV() for n_neighbors was 32.

#### 2.7.5. Logistic Regression

Logistic regression is a very simple classification model, which can be extended to a linear binary classification model [74,75]. Therefore, to obtain the classification and its probabilities of belonging to a certain class, this model was implemented as a suggestion. The range of values used in the grid search to find the most optimal value of C was: 0.001, 0.002, 0.01, 0.02, 0.03, 0.1, 0.2, 0.3, 0.4, 1.0, 2.0, 3.0, 4.0, 5.0, 10.0, 20.0, 30.0, 40.0, 50.0, 60.0, 70.0, 80.0, 90.0, 100.0. To really predict the probability that a certain sample belongs to a specific class, the so-called logistic sigmoid function (reverse logit) [74,75] is employed, which is between the ranges [0, 1].

The most optimal hyperparameters to construct the logistic regression algorithm formed with 16 features are C = 0.2 and random_state = 42. Thus, the most optimal value for the regularization parameter (C) is 0.2 and 0.002 for 16 and 8 features respectively. This parameter makes it possible to find a good trade-off between bias and variance through complexity tuning. For this situation, increasing the regulation force (small values of C) allows finding that good compensation, however, by decreasing the regulation force, there are no significant changes in reducing over-learning or under-adjustment. Figure 6c and Figure 7c show the validation curve for the range of values defined for C. When increasing or decreasing the regularization force, there is no significant difference in algorithm precision. It should also be noted that over-learning is almost nil, both when increasing or decreasing the force of regularization.

Ranganathan et al. [74] indicates some situations to be taken into account when using regression logistic, such as selecting the appropriate input variables. Other consideration in this context implies the use of too many variables in the pretense that better results can be obtained, which can cause a decrease in real associations and cause substantial errors with wide and imprecise confidence intervals, or even not real associations. Conventional technique consists of first running the univariate analysis (the relationship of the result with each predictor, one at a time), then using only those variables that meet a predetermined limit of significance to run a multivariate model or, those variables with greater importance, as in this case. Avoiding the use of highly correlated variables can help improve the performance of these algorithms. Moreover, if multicollinearity exists, i.e., input variables are highly correlated with each other, the impact of these variables becomes less accurate. The relationship between the predictor variables and the dependent variable is uniform in the regressions schemes, which in turn can be positive or negative, linear or non-linear, where at the same time remains constant in the different values. Nevertheless, this conjecture may not be correct for particular associations; e.g., mortality from COVID-19 may be higher at age extremes. Accordingly, obtaining COVID-19 mortality predictions by means of age will not yield meaningful results if ages ranges from small values to high values. Also, regression approaches formulated from a determined groups of patients may not be ideal to patients in other context. In this sense, this type of technique must handle continuous input variables in the appropriate way; for instance, for continuous data such as age, it maybe common to divide this data into categories, which is not a good idea, since the limits may be arbitrary and a part of the data is lost.

#### 2.7.6. Artificial Neural Network

This alternative model ha been designed to develop a simpler model and avoid some overfitting problem to improve accuracy. Also, if probability returned by the neural network is greater than 0.50(50%), the patient is classified as recovered patient; while if probability is lees than 0.50, then, the patient is classified as dead.

**Figure 8:**
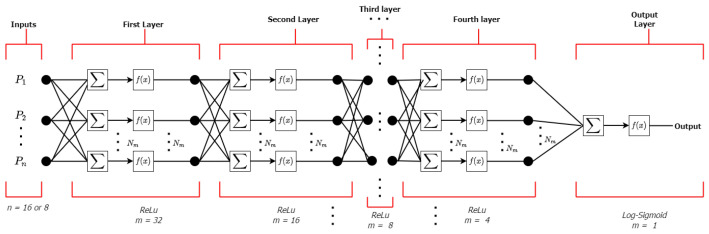
Artificial neural network architecture.

For this network, Adam algorithm was chosen as the optimizer in the training phase. What this module does is optimize the parameter values to reduce the error made by the network. The process by which this is done is known as backpropagation. The term backpropagation refers only to the method by which the necessary gradient is calculated to adjust the parameters of the network. It is an optimization method based on the descending gradient to gradually adjust the weights of the neural network and train it to do what we want, being the traditional training method [76]. The learning rate employed in training phase was 0.001; while the number of training epochs was 75. Besides, accuracy was used as performance metric meanwhile the minimization of the binary_crossentropy cost function was reached. The chosen model type was Sequential(), and for all layers, the Dense() layer type was selected. Finally, the weights were initialized using kernel_initializer = “uniform”. Figure 8 shows the architecture of the neural network, which is composed of an input layer (with 16 or 8 neurons according to the appropriate model), 4 hidden layers with 32, 16, 8 and 4 neurons, as well as an output layer with 1 neuron. For hidden layers, a ReLu activation function was implemented, while for the last layer a sigmoid function was used [75,77,78]. The number of epochs performed and the number of neurons used per layer allowed to obtain the most optimal models, achieving a good bias-variance compensation. The configuration of these two hyperparameters improves the convergence of the algorithm, which allows the weights to be learned more quickly.

#### 2.7.7. Majority Voting

Other technique utilized was the majority voting principle. This means that the class label that has been predicted by the majority of the classifiers was selected, i.e., this label has received more than 50% of the votes [79]. To form the majority voting scheme, 3 different algorithms were applied as predictors: KNN, logistic regression and decision tree. These parameters were selected by searching grids. The idea is to build a more complex model from simple models, such as KNN, logistic regression and decision trees, which allow to improve the performance of the algorithms, obtaining more reliable and precise classifications, in addition to improving the sub-adjustment in the algorithms. The most optimal hyperparameters to construct the logistic regression algorithm are C = 0.004 and random state = 42. The most optimal hyperparameters to build the algorithm were k neighbors = 31 and *p* = 2. These parameters are obtained by grid search. The most optimal hyperparameters to build the algorithm version consists of max depth = 11, criterion = Gini and min samples split = 2. In this sense, voting = soft is also the most optimal hyperparameter obtained for majority voting model.

There is a problem in relation to the underfitting data from the different implemented algorithms (KNN, ANN, majority vote, etc.), data collected has some possible bias or noise due to an erroneous data collection or there are records with non-representative and/or false information. For the majority vote technique, principal component analysis was also used [80], implemented by using the sklearn library. The value of the parameter n_components = 0.95 indicates that the explained variance is between 95% and 99%, which compresses the dataset *n* components less than the original.

### 2.8. Metrics

The purpose of this project is to predict with the highest precision the outcome of a particular patient depending multiple factors. Therefore, in order to evaluate the model, 5 evaluation metrics were considered for this study. The following terms are used in the equations: TP, true positive; TN, true negative; FP, false positive; and FN, false negative.

#### 2.8.1. Accuracy

Prediction error and precision provide general information about the classification errors of some samples, according to Equation (2) [42].

(2) $$ERR=\frac{FP+FN}{FP+FN+TP+TN}.$$

#### 2.8.2. Precision

Precession is an important metric to identify the number of correctly classified patients in a dataset that contains a number of unbalanced classes, following Equation (3) [42].

(3) $$Precession=\frac{TP}{FP+TP}.$$

#### 2.8.3. Recall

As well, recall is an important metric to identify the number of correctly classified patients in a dataset that contains a number of unbalanced classes, according to Equation (4) [42].

(4) $$Recall=\frac{TP}{FN+TP}$$

#### 2.8.4. *F*1 Score

*F*1 score achieves the perfect balance between precision and recall, thus providing a correct assessment of the model’s performance in classifying COVID-19 patients, according to Equation (5) [42].

(5) $$F1=2\times \frac{precession\times recall}{precession+recall}.$$

#### 2.8.5. Hyperparameters Optimization

For optimal performance of the presented models, a search was performed on a grid of chosen parameters to obtain a set of best-performing parameters. The grid search was implemented using the GridSearchCV() function from the scikit-learn library [42,81,82]. The optimization and choice of hyperparameters is also based on different techniques such as optimization for genetic algorithms, Bayesian optimization and for machine learning [83,84]. Subsequently, validation and learning curves were used to diagnose and address under-adjustment or over-learning problems. Finally, the cross-validation of k iterations was used in a nested way with the grid search in order to estimate the performance of the model with data that has not been seen previously [85,86].

#### 2.8.6. Cross Validation

The training dataset was randomly divided into k = 10 iterations without replacement, where k − 1 iterations were used for model training and one iteration for performance test. This process was repeated k times to obtain k models and performance estimates, This was combined with the grid search technique [85,86]. The GridSearchCV() function for the exhaustive search of specified parameter values for an estimator was defined with cv = 10. This parameter indicates the number of folds (k iterations and models) for cross-validation in K-Fold when looking for the best n parameters of the estimator.

## 3. Experiments and Results

The preprocessed dataset described in the previous sections (see Table 3) have been used to train multiple AI classification models. F1 Score was chosen as an additional metric for comparison, and it was chosen as the main decision metrics; F1 score is commonly used when the classes are imbalanced and there is a serious downside to predicting false negatives, as in our case. For example, if we use our model to predict whether or not a patient recovers, false negatives are really bad (e.g., predicting that a patient is not likely to recover, when in fact he or she is), thus F1 score will penalize schemes that have too many false negatives more than accuracy will.

The label “1” was assigned to the surviving patients and the label “0” has been established for the deceased patients. Otherwise, a value of k = 10 (folds) was used for cross-validation, since empirical research indicates that this value allow to find the best trade-off between bias and variance on large subsets.

### 3.1. Implementation Details and Runtime

Table 4 details the execution environment for each implemented model. Here is also considered cross-validation time and application of the grid search technique. Although the ANN computing time is greater, its corresponding training and testing phase was accelerated by GPU; while the other algorithms were trained and validated using the CPU as execution environment. In these cases, an off-line model training can accept this type of runtime since the online test process is only one sample per period. The different phases of feature extraction, training and testing for the different models were run on a Dell XPS 9550 series (Intel(R) Core(TM) i7-6700HQ CPU @2.60 GHz with 16 GB 2666 MHz DDR4 RAM), which was manufactured by Compal in Kunshan, China; while the training of the ANN model was accelerated using an NVIDIA GeForce GTX 960 M card (containing 2 GB of GDDR5 RAM, memory clock of 2500 MHz and 640 processing cores), which is manufactured by Taiwan Semiconductor Manufacturing Co., in Hsinchu, Taiwan. The CUDA 11.0 library has been used in Windows 10 to provide access to the GPU-based matrix operations.

### 3.2. First Data Cut Results (December 2020)

This analysis of results is considered until 15 December 2020. Our research focuses primarily on the analysis to date, later this analysis focuses on future data cuts to assess the behavior of our suggested algorithm during the evolution of the pandemic.

#### 3.2.1. Feature Selection with Random Forests

Based on the number of correct predictions per class, the model tends to make a greater prediction error for deceased patients. While to predict recovered patients, the algorithm has a better performance, since it makes fewer incorrect predictions. The results obtained by the estimator for the training and testing subsets are shown in Table 5. First of all, random forests was applied as feature selector, and later as a initial predictor. This is the reason why the model obtains best metrics with 8 features than with 16. Results obtained give notion of the accuracy on unseen data, which shows that this model has good performance with distinct data; thus, this algorithm has little variance in accuracy on data unknown to the algorithm.

Given to the slight class imbalance, a good guiding metric is F1 score. This algorithm obtains an precision of 0.889 on the training subset, and for the testing subset it has a precision of 0.886 with 16 features. The algorithm presents few overfitting, which indicates more reliable predictions. The precision obtained by the algorithm shows the efficiency to obtain high performance even in subsets with an unbalanced class size. The model obtains an F1 score of 0.8629 for the training subset, while a F1 score of 0.8602 for the testing subset based on 8 features (its best model), this implies the algorithm can differentiate the classes with a high confidence of 86%, and learns to detect the recovered patients class in 86.29% of the cases. There is a bias in both classes (deceased and recovered), in this situation, the class of deceased patients is the most biased. This not only makes it an ideal model to predict mortality, it is also ideal for reliably, obtaining the features importance in the association of death from COVID-19. The accuracy is 86.29% for the training subset, while for the testing subset is 86.02% in the best model (8 features). When the algorithm predicts deceased and recovered patients, it classifies 86.02% of the total samples correctly and it learns to guess them correctly in 86.29%. This generally means that the classifier is doing a good job (for both subsets) in identifying the patients health status, it obtains a good number of correct positive predictions (real deceased and real recovered patients).

For the training and testing subset, the algorithm has a recall of 0.8639 and 0.8612, respectively. That is, this model correctly identifies 86.12% of recovered patients and learns to identify them in 86.39% for the best model (8 features). Improving precision generally reduces recall, and vice versa, as is this case. precision and recall provide important information about the quality of the models, indicating whether there is a bias towards positive or negative values.

The results show that the developed algorithm can accurately predict the risk of mortality in COVID-19 patients based on risk factors in the patients. Also, comparing the training and testing results shows that the algorithm has low variance, since the difference of performance in both subsets is very subtle. These indices of low variance indicate that the algorithm is capable of generalizing to solve unseen situations, that is, the algorithm is capable of applying what it has learned with new data (probably with different patterns) making reliable predictions.

#### 3.2.2. Decision Tree

Results in this scheme indicate that the model will not have much variation in their performance with unknown data and will give reliable predictions in different situations. The results obtained for this estimator are presented in Table 6. The model accuracy for the training subset is 86.4%, and 85.9% for the test subset with the best model (8 features). In other words, the model correctly predicts 85.9% of the total samples and learns to correctly classify 86.4% of the total samples. As a result, these scores indicate that the algorithm presents a good performance over the total samples (positive and negative predictions), with almost zero overfitting. But these scores do not provide additional information on the individual performance of the classes (deceased and recovered), that is, it does not answer the question, does the algorithm have the same performance for both classes?

In training and testing subset the model obtains an precision of 90.19% and 89.81%, respectively, which are good performance results with 16 features. This means a low estimation bias in the recovered patients class (approximately 10%), due to a high percentage of positive cases detected, and during the learning, this model has a slight bias towards the class of true positives. This model maintains an accuracy and precision very similar to the random forest model, however, the complexity of the model is high, due to its depth, which as a guide in clinical centers can become impractical.

The classifier has an acceptable ability to discriminate positive cases from negative ones (the proportion of positive cases that were correctly identified by the algorithm). Regarding the recall metric, this model learns to identify 83.1% of recovered patients (in training subset), while for the test subset, 82.5% of recovered patients are identified with 8 features. An F1 score of 86.2% is obtained for the training subset, while in test subset a score of 85.7% is obtained with 8 features. In specific, the model presents high precision and low recall: the algorithm does not detect the class very well, but when it does, it is highly reliable, it learns to detect the recovered patients class in 86.2% and it manages to identify 85.7% of the recovered patients. Furthermore, the algorithm exhibits good performance and a slight bias on unbalanced classes.

For certain types of contexts, this basic classifier can be very effective. However, in this case it is inefficient due to its complexity and not for its performance, making it an impractical model in clinical practice; so it is necessary to search for other more robust classification schemes. Underfitting phenomenon occurs when the algorithm fails to find a relationship between the learning data, failing to make good predictions. Commonly, overfitting is present while using neural networks. During learning, the aim is to minimize prediction errors by performing a certain number of learning loops (iterations). The algorithm will learn from your mistakes with each loop and correct itself.

#### 3.2.3. Logistic Regression

Table 7 presents the results of logistic regression algorithm. The precision for the training subset is 0.8853, while the recall score is 0.8387 with 16 features. When predicting the health recovery of a patient, the algorithm is correct 88.53% of the time and learns to identify recovered patients in 83.87%. Therefore, these scores indicate that the model has a high performance in identifying positive predictions as really positive (high accuracy), and it misclassifies some positive examples as negative (moderate completeness). Otherwise, the precision for the test subset is 0.8747, and the recall score is 0.8314 with 16 features. This algorithm has an acceptable ability to identify recovered patients in 83.14% of cases, and it is correct with a confidence of 87.47%, that is, the algorithm is very efficient and reliable in its predictions.

As was discussed, the F1 metric shows a big picture of performance, based on accuracy and completeness. During training, the model does not learn to detect the recovered patient class very well, but when it does, it is highly reliable, obtaining an F1 score of 0.8553 with 16 features. When it is predicting a recovered patient, it identifies it in 85.53% of cases very reliably. For unseen data, the model obtains the same performance in testing subset, a F1 score of 0.8525 (it correctly detects 85.25% of the total samples in recovered patient class); having a slight bias. This model is able to predict recovered and deceased patients with the same efficiency (without memorizing the data). In general, for the corresponding training and testing subset, the performance of the model (accuracy) of correct predictions is 0.8550 and 0.8528 with 16 features, respectively. That is, when this model predicting the recovery of a patient, the algorithm correctly identifies 85.28% of the total samples and learns to correctly identifies 85.50% of all samples; this model achieves high performance, thus the classifier has the same generalization ability for seen and unseen data.

Table 7 suggests to improve the models performance, where it is necessary to take additional characteristics, such as vital signs, severity status, type of viral strain, clinical features, among others. To reduce the percentage of error, it is also necessary to use a more robust and homogeneous dataset (with additional characteristics), so it would be possible to avoid underfitting and significantly increase their performance. In addition, to obtain better results, it is necessary to implement other models such as neural networks and majority voting, which have greater robustness. The simplicity of this model can make it practical in real clinical situations, besides, it provides additional information giving the results in terms of probabilities.

#### 3.2.4. Knn

Results in this scheme show that the algorithm has not performance variations with unseen data and there is not data storage by the model. Table 8 shows the results for the different evaluation metrics on the training and testing subset. The algorithm obtains an precision of 0.8796 in the test subset, while in the training subset it achieves an precision of 0.8853 with 16 features; i.e., the model correctly identifies 87.96% of the positive true and learns to identify with a reliability of 88.53%. It can be seen that the model obtains few false positives (high accuracy), so this means that the algorithm erroneously classifies recovered patients as deceased very rarely. Otherwise, the classifier reaches a recall in the training subset of 0.8387 with 16 features, that is, KNN learns to correctly identify 83.87% of the recovered patients. For the testing subset, this model achieves a recall of 0.8320, so it correctly identifies 83.20% of the deceased patients. It means an acceptable number of false positives (moderate recall). Thus, it can be seen that the algorithm predicts a considerable number of erroneously recovered patients (classified as deceased).

An F1 score of 0.8614 is obtained in the training phase, as well as 0.8552 in the testing phase with 16 features. It means that the model correctly detects 85.51% of the deceased patients, and the model learns to acceptably distinguish one class from the other with a high reliability in its predictions of approximately 86.11% in recovered patient class. This learned pattern is applied effectively to data not seen by the model. Unlike other models, this algorithm better distinguishes samples between classes, but with less reliable predictions. Finally, a accuracy of 0.8558 is achieved in testing phase with 16 features, which represents a high capacity of the model to obtain correct predictions; i.e., the model correctly predicts 85.58% of the total samples in testing. While in training KNN, learns to correctly classify 86.16% of the total patients.

#### 3.2.5. Artificial Neural Network

Results shown in Table 9 validate the accuracy obtained in the importance and selection of features. When building a less complex model, discarding irrelevant features, inferior predictions are not obtained, this validates the good performance of the random forest model for feature selection. Furthermore, it demonstrates the high efficiency of AI models to solve complex prediction problems. However, the application of more robust and complex techniques was not the solution to the underfitting problem. This algorithm confirms that age, pneumonia, diabetes, requiring intubation and/or other complications are potential predictors of death from COVID-19.

For training subset, a precision of 0.8810 were obtained with 16 features, i.e., the model learns with high confidence in its predictions (it is correct 88.10% of the time). The recall metric has a result of 0.8524 (in training) with 8 features, learning to identifies 85.24% of the patients. Besides, an F1 score in training phase of 0.8644 is obtained with 16 features, i.e., this classifier correctly and reliably detects 86.44% of recovered patients. This means that the model manages to differentiate one class from the other quite well, with an acceptable reliability due to it includes samples of the other class. Addition, in training subset, our model has an accuracy of 0.8644 with 16 features; in other words, when it predicts a recovered or decreased patient is correct 86.44% of the time.

For testing subset, this model has a precision of 0.8786 with 16 features (that is, it is correct 87.86% of the time in the positive class). For recall, a score of 0.8493 is obtained with 8 features, so it detects 84.93% of recovered patients; and for F1, a score of 0.8602 is achieved with 16 features (harmonic mean between precision and recall), in other word, this model correctly identifies 86.02% of recovered patients. These three metrics can be interpreted as high confidence that positive predictions (actual recovered) are actually positive (high precision), with some positive examples misclassified as negative (moderate recall): recovered patients detected as deceased patients. Also, the algorithm is very efficient on balanced datasets. In testing subset, our classifier has an accuracy of 0.8636 with 16 features, that is, when our model predicts a recovered or decreased patient, it is correct 86.36% of the time.

These results affirm the need to use a more robust and homogeneous datasets in order to reduce the error rate. It is necessary to find other factors highly related to the death of SARS-COV-2 to improve the performance of the models and build more reliable predictors. In addition, optimizing and improving current techniques can help improve the performance of models. These results are good indications that it is possible to build alternative predictors to the existing ones. Finally, the results also demonstrate that artificial neural networks are an adequate and robust model to accurately predict the risk of mortality, mainly due to the fact that the best f1 score was obtained with them.

#### 3.2.6. Majority Vote

The building of a more complex model from simple models, such as KNN, logistic regression and decision trees, it is not a technique that can improve or decrease algorithm performance. This could be due to noise in data collection or the creation of new features, since the more complex models maintain the same performance compared to the simple schemes.

Table 10 shows the results for this model, where an accuracy of 0.8625% in training and 0.8574% in testing is obtained with 8 features. This means that our classifier correctly predicts 85.74% of the total samples of both classes and learns to classify 86.25% of the total patients independently of the classes. The COVID-19 patient health status classifier is doing a good work of identifying deceased and recovered patients without suffering from overfitting problems. The implemented model has an precision of 0.8916 with 16 features, this means that when it predicts a recovered patient, it is correct 89.16% of the time in training phase. On unseen data (in testing subset), the classifier predicts 88.75% of the positive classes. In terms of recall effectiveness, when it learns (training subset), the model has a value of 0.8357 with 8 features; thus, the algorithm correctly identifies 83.57% of deceased patients. In the testing phase, it correctly identifies 82.82% of the recovered patients. These results can be summarized in a single metric (F1 score), with scores of 0.8617 (in training) and 0.8559 in the testing phase with 8 features. These metrics can be interpreted as: the model correctly and very reliably detects 85.59% of deceased and recovered patients, and during training, the algorithm learns to correctly classify 86.17% of patients.

These results indicate that the technique of a meta-classifier does not allow to obtain a more generalized performance than each classifier separately, in this context. In other words, this technique does not allowed to get more precise and robust predictions than independent classifiers, so it is not a technique that optimizes prediction performance. It is concluded that age, suffering from pneumonia, predict complications, suffering from certain diseases such as diabetes and hypertension become the most vulnerable population (with a higher risk of death), due to they have poor physiological and immune responses to viral situations. In addition, immune cells lead to poor suppression of viral replication, causing immune dysfunction as described in studies already carried out.

#### 3.2.7. Models Comparison

**Figure 9:**
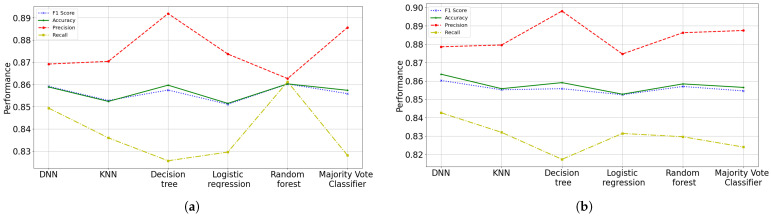
Comparison of the models performance (first data cut). (**a**) 8-features based; (**b**) 16-features based.

The study shows that decision tree algorithm performs (strictly) better in predicting recovered patients from SARS-COV-2 in terms of precision with 8 and 16 features (see Figure 9). Due to the dataset is slightly unbalanced and a false negative is a crucial problem, the F1 score is the best metrics in this case; hence artificial neural network is a better balanced model in prediction in our context with 16 features (with a F1 of 0.8602 in test and 0.8644 in training). Reducing the number of features, we can obtain models with less complexity (using the most influential features), but they do not necessarily give us better results in degree of confidence. Nevertheless, the features importance selection allowed the 8-feature random forests model to achieve a score equal to 16-feature ANN, although the learning capacity of the neural network came out better during training.

The different algorithms developed have high precision and moderate recall. Otherwise, the classifiers do not detect the recovered patients class very well, this does it slightly biased, but when this does it, their predictions are highly reliable. It can also be seen that in the algorithms where the identification capacity of the recovered patient class (recall) is increased, the reliability of the predictions (precision) decreases, such as 8-feature KNN, ANN in both models and 16-feature logistic regression. Contrary, in algorithms where the detectability of the recovered patient class decreases (recall), the reliability of the predictions (precision) increases, such as decision trees and majority vote classifier in both schemes. The ANN model seems to be the best option, not only because it has good performance and low overfitting compared to the other models, also because it allows to obtain the classification in terms of probabilities with a slight difference in performance compared to the other implemented models. Having the probabilities of risk of death or healing, it gives more detailed and precise information on the health status of patients, thus this provides an important diagnostic tool.

### 3.3. Second Data Cut (October 2021)

**Figure 10:**
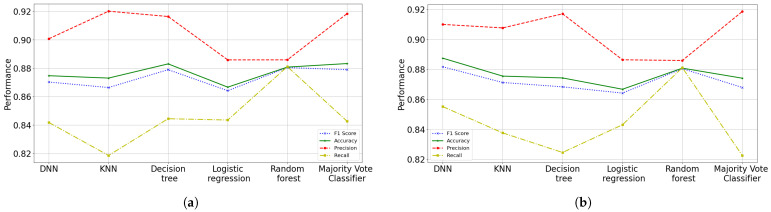
Comparison of the models performance (second data cut). (**a**) 8-features based; (**b**) 16-features based.

In this dataset it is assumed that there are unvaccinated people and people with at least one dose of vaccine. This model has a slight increase in performance compared to previous models, since it learns to better identify recovered patients. An F1 score of 0.8846 is obtained for the ANN training subset (the best model) with 16 features, while in the testing subset, a score of 0.8816 is obtained. Briefly, the model has high precision and low recall (a bias towards the class of deceased patients). The algorithm does not detect the class very well, but when it does, it is highly reliable; it learns to detect the deceased class in 88.46% and when predicting, it manages to identify 88.16% of the deceased patients (see Figure 10).

### 3.4. Third Data Cut (22 February)

**Figure 11:**
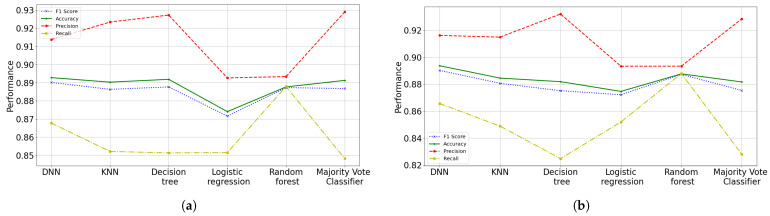
Comparison of the models performance (third data cut). (**a**) 8-features based; (**b**) 16-features based.

In this dataset, it is assumed that there are unvaccinated people and people with up to two doses of vaccine. It is observed that this scheme has better performance than the previous models, since it learns to better identify recovered patients and with better reliability. An F1 score of 89.11% is obtained for the ANN training subset (the best model), while a value of 89.01% is obtained in testing with 16 features. In other words, the model presents high precision and low recall (see Figure 11). Our model (in the testing subset) has a recall of 0.8656, i.e., it correctly identifies 86.56% of all recovered patients. Besides, our model has a precision of 0.9161 in testing phase, namely, when it predicts that a patients has healed, it is correct 91.61% of the time. This model does not present overfitting, however, it does present underfitting.

### 3.5. Fourth Data Cut (April 2022)

**Figure 12:**
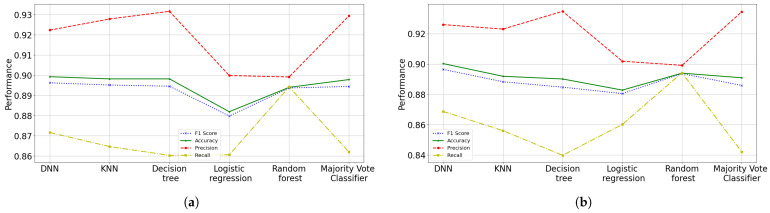
Comparison of the models performance (forth data cut). (**a**) 8-features based; (**b**) 16-features based.

It can be assumed that in this dataset there are people unvaccinated and people with up to a third booster dose. Figure 12 presents the results obtained in the 8-feature and 16-feature testing phase comparison. It can be seen that the classifier obtains a stable performance in the evolution of the pandemic, considering this data cut as the most recent modeling. In addition, this model presents a decrease in underfitting and does not present overfitting. It is observed that this model has similar performance to previous model, since it learns to identify deceased patients and with good reliability. In testing phase, the best model (16-feature ANN) has a recall of 0.8687, namely, i.e., it correctly identifies 86.87% of all recovered patients. And our model has a precision of 0.9260, i.e., when it predicts a recovered patient, it is correct 92.60% of the time. Moreover, an accuracy of 0.9002 is obtained, or 90.02% correct predictions; while an F1 score of 89.76% is obtained for the training subset, as well as a F1 score of 89.64% is obtained in test phase. In other words, the model presents high precision and low recall.

### 3.6. Comparison with Related Works

**Figure 13:**
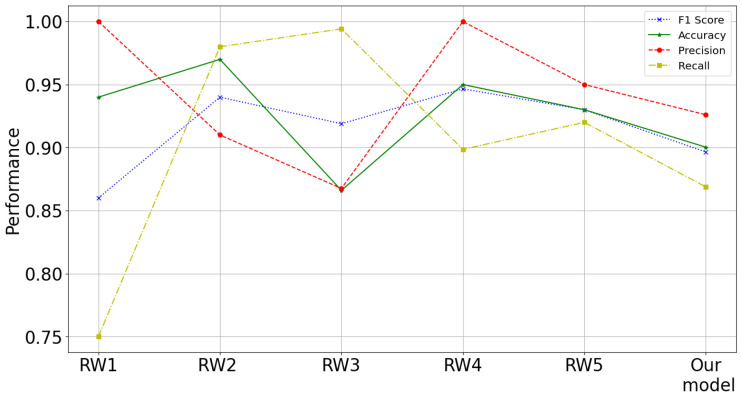
Comparison between the-state-of-the-art (RW1 [42], RW2 [44], RW3 [53], RW4 [55], RW5 [45]) and this research.

A metric comparative regarding other research is shown in Figure 13, in which values for each indicator provides the corresponding visual description about the particular data and context. For instance, we can find a contrast for large values of precision, but small values of recall [42], having a regular F1 score with its balance metric; i.e., the detectability of the appropriate class decreases (recall), while the reliability of the predictions (precision) increases. Nevertheless, we can find a stable model with a better distribution of its metrics [55], in which large values for precision and recall were obtained, thus having outstanding values for F1 (close to 94.5%). On the other hand, we can find small values for precision with large values for recall [53]; i.e., the identification capacity of the appropriate class (recall) is increased, while the reliability of the predictions (precision) decreases. In this sense, our 16-feature ANN model, whose metric values are 90.02% in accuracy, 89.64% in F1, 92.60% in precision and 86.87% for recall, maintain indicators that are not as high as the other models, but are stable in their various metrics. Do not forget that each work uses different variables in varied environments with particular circumstances, as well as in their own geographical areas with different populations.

## 4. Discussion

Early identification of patients at risk of die has several predictable benefits including better allocation of critical care staff and supplies, the ability to contact people who are not currently admitted to the hospital and ensure they are thoroughly evaluated; and the ability to relocate inpatients to a facility capable of providing a higher level of care. Our designs aim to be trained first to identify the most relevant characteristics and then predict patient mortality. Initially, simple linear models were built working with the 16 or 8 parameters of the epidemiological study, but in some models or cases we sought to simplify the model through excluding features, to increase its performance and reduce underfitting.

The benefits of this research is the identification and assessment of new features for prediction models of health status. And given the results, it can be seen good indications showing that it is possible to achieve alternative models that are alike efficient based on quick diagnoses. Our predictive model differs from others in several ways, first of all, this makes it possible to predict the health status of a patient from a simple and faster medical diagnosis in the point of view of users (by using the mobile app, for instance). By not analyzing variables such as vital signs, patient region, blood oxygen, among others, this way allows faster medical assessments to identify patients with more susceptibility to die. Second, it proposes a model taken to a higher level of abstraction with a performance similar to other existing models, which is independent of specific cases where the region of the patient is taken into account; this is achieved by considering different variables of prediction. Finally, this analysis uses a large amount of data from different regions of a single country (Mexico). Another strength is the use and validation of widely used prediction models in different circumstances. Also the application and comparison of different methods allows to identify the appropriate techniques in the prognosis of the health status of patients infected. In addition, this research allows the identification of new techniques that could be useful; the identification of existing variables that have not been applied until now (as far as is known) in predicting SARS-COV-2 mortality.

This study has several considerations, for example, it is a retrospective study carried out in a single country in different health institutions. This work is a first step towards AI-assisted clinical prediction. Our predictive model needs to be externally validated using multi-institutional data and/or prospective studies from other countries before clinical application can be made. If our tool is externally validated in due course, then we imagine that this predictive model can be used by frontline people to more effectively diagnose COVID-19 patients. The validation of these mortality predictors in future prospective studies could be useful to identify and stratify the risk groups, as well as to provide the necessary health services.

In addition, this study is under different circumstances and has been carried out in different regions of Mexico. These circumstances may differ from place to place, causing a different pattern in the statistics from one place to another. They may differ in terms of the quality of medical care in the country (hospital infrastructure, quality of care, professional training, among others), in addition to factors such as obese population, the quality of their diet, the average age, the quality of health (diseases suffered by age), the number of people infected by age, the population’s habits (smoking), to mention a few. In this sense, we only analyzed clinical variables at patient admission (risk factors). A final comment can be aimed at emphasizing that the circumstances in which COVID-19 occurs are unusual and are constantly changing. Thus, the number of hospitalized patient and their flow in ICU, as well as the mortality factor may depend on the region load and the available resources of individual hospitals, which also differ between countries, as well as other patterns and behaviors in habits and characteristics of health in the population by region.

## 5. Conclusions and Future Work

The current application of data analysis techniques around world is more and more necessary today in order to propose models to process patient data seeking to define efficient treatment strategies. In this work, a comparison of machine learning techniques was made with the aim of determining the probability that a person infected by COVID-19 can recover. We presented different prediction models, where the ANN scheme has been chosen as the most accurate framework, because not only we can obtain the patient’s classification, because also it can be obtained in terms of probabilities. Although the different proposed and implemented prediction models do not present an overfitting problem, they do present a slight underfitting problem. These models present a low variance, but a high bias, and the models underfit the data since they do not achieve a high level of abstraction of them. Since the parameters in all the models were adjusted and regularized as best as possible by means of grid searching and among other techniques, it is possible that more features may need to be built or collected to improve the accuracy of the models. Although a process of determining the features importance was done, like many models, selecting more features gave better results.

As a solution to improve the accuracy and precision of performance and reduce the under-adjustment of the algorithms, it is suggested to collect more characteristics, since there may be more related risk factors (not considered) in determining the mortality of patients infected with COVID-19. Due to that, there may be other different and important factors (with high influence and relationship) to determine more accurately whether a patient infected can survive or die. Such factors can be type of viral strain, seriousness and professional capacity of the medical staff (experience, physical exhaustion, preparation); besides, some symptoms or vital signs can be included, e.g., the amount of oxygen in the patient’s blood.

Otherwise, the data analyzed in this study have revealed that the rates (probabilities) of mortality were higher if the patients presented the following main risk factors: requiring intubation, having pneumonia, advanced age, diabetes and hypertension. For the selection of these characteristics, a random forest model was implemented to identify the features importance, obtaining an accuracy of 89.40% with a F1 metric of 89.37% in testing phase. It was also found in this research that people with pneumonia and advanced age are not only have more possibilities to die, but also to have more complications (be intubated or entering the ICU) due to COVID-19. Finally, at an older age there is a higher risk of suffering from risk factors associated with death. Therefore, the performance error of the different implemented algorithms is around 14% or less. ANN was the proposed model to predict the risk probability of death or healing of patients with COVID-19, obtaining an accuracy of 89.8%, a precision of 92.3%, a recall of 86.8% and F1 score of 89.5%. For this work, an open data set “COVID-19 databases in Mexico” provided by the Secretary of Health of Mexico was used, which was filtered to be adapted to the requirements.

In a future work, the construction of a new more robust dataset for the training of these models is suggested. A more representative and robust dataset will reduce the under-adjustment in the algorithms, finding in a more precise way the importance of risk factors. In addition, the improvement of the employed techniques and the implementation of new schemes and models are suggested.

## Figures and Tables

**Table 1:** Description of the datasets.

Data Cut (Dataset)	Recovered Patients	Unrecovered Patients	Total Patients with COVID-19
15 December 2020	122,311	116,487	238,798
October 2021	209,317	207,245	416,562
February 2022	239,814	237,440	477,254
April 2022	327,183	323,944	651,127

**Table 2:** Dataset variables with higher correlation.

	Death	Intubated	Pneumonia	Age	Diabetes	Hypertension	ICU
Death	1	0.692	0.675	−0.565	0.289	0.311	0.651
Intubated	0.692	1	0.934	−0.490	0.271	0.277	0.927
Pneumonia	0.675	0.934	1	−0.470	0.262	0.264	0.885
Age	−0.565	−0.490	−0.470	1	−0.291	−0.383	−0.445
Diabetes	0.289	0.271	0.262	−0.291	1	0.404	0.248
Hypertension	0.311	0.277	0.264	−0.383	0.404	1	0.249
ICU	0.651	0.927	0.885	−0.445	0.248	0.249	1

**Table 3:** (%) Dataset features importance.

Feature	Importance	Feature	Importance
SEX	0.217	PNEUMONIA	34.522
INTUBATED	47.702	AGE	14.891
PREGNANCY	0.142	DIABETES	1.140
EPOC	0.099	ASTHMA	0.016
INMUSUPR	0.007	HYPERTENSION	0.770
ANOTHER_COM	0.037	CARDIOVASCULAR	0.048
OBESITY	0.038	CHRONIC KIDNEY	0.331
SMOKING	0.016	ICU	0.015

**Table 4:** Models runtime (seconds).

Model	First data cut		Second Data Cut		Third Data Cut		Fourth Data Cut	
	Training	Testing	Training	Testing	Training	Testing	Training	Testing
ANN-8	913.338	2.697	1335.154	5.101	1570.937	5.935	2820.562	12.383
ANN-16	873.773	2.325	2698.276	10.599	4751.317	11.008	3364.989	12.536
KNN-8	154.104	2.780	188.100	11.156	177.341	10.228	208.685	12.496
KNN-16	272.315	4.795	249.878	14.660	285.668	17.766	303.810	17.203
R. forest-8	432.060	0.151	976.704	0.321	1270.880	0.317	1751.717	0.305
R. forest-16	754.794	0.109	1266.460	0.199	1467.805	0.323	1353.237	0.417
L. reg-8	73.342	0.001	27.956	0.004	25.941	0.004	12.496	0.005
L. reg-16	109.610	0.001	67.622	0.018	82.030	0.006	82.030	0.006
M. vote-8	29.410	1.405	50.446	16.945	35.544	9.543	41.719	13.307
M. vote-16	57.986	2.374	46.430	10.549	74.721	0.009	102.782	18.150
D. tree-8	40.560	0.015	30.289	0.041	33.106	0.029	35.737	0.026
D. tree-16	41.724	0.1	37.809	0.0189	45.717	0.027	49.398	0.027

**Table 5:** Random forests metric results for the first data cut.

	Accuracy		F1 Score		Precision		Recall	
	Training	Test	Training	Test	Training	Test	Training	Test
R. forest-8	0.8629	0.8602	0.8629	0.8602	0.8650	0.8626	0.8639	0.8612
R. forest-16	0.8623	0.8584	0.8614	0.8570	0.8895	0.8863	0.8350	0.8296

**Table 6:** Decision tree metric results for the first data cut.

	Accuracy		F1 Score		Precision		Recall	
	Training	Test	Training	Test	Training	Test	Training	Test
D. tree-8	0.8642	0.8597	0.8626	0.8575	0.8958	0.8919	0.8318	0.8257
D. tree-16	0.8637	0.8591	0.8609	0.8558	0.9019	0.8981	0.8235	0.8173

**Table 7:** Logistic regression metric results for the first data cut.

	Accuracy		F1 Score		Precision		Recall	
	Training	Test	Training	Test	Training	Test	Training	Test
L. reg-8	0.8539	0.8515	0.8541	0.8511	0.8749	0.8737	0.8342	0.8296
L. reg-16	0.8550	0.8528	0.8553	0.8525	0.8853	0.8747	0.8387	0.8314

**Table 8:** KNN metric results for the first data cut.

	Accuracy		F1 Score		Precision		Recall	
	Training	Test	Training	Test	Training	Test	Training	Test
KNN-8	0.8578	0.8524	0.8579	0.8528	0.8792	0.8704	0.8377	0.8360
KNN-16	0.8616	0.8558	0.8614	0.8552	0.8853	0.8796	0.8387	0.8320

**Table 9:** ANN metric results for the first data cut.

	Accuracy		F1 Score		Precision		Recall	
	Training	Test	Training	Test	Training	Test	Training	Test
ANN-8	0.8613	0.8588	0.8616	0.8591	0.8710	0.8692	0.8524	0.8493
ANN-16	0.8644	0.8636	0.8644	0.8602	0.8810	0.8786	0.8485	0.8426

**Table 10:** Majority vote metric results for the first data cut.

	Accuracy		F1 Score		Precision		Recall	
	Training	Test	Training	Test	Training	Test	Training	Test
M. vote-8	0.8625	0.8574	0.8617	0.8559	0.8893	0.8856	0.8357	0.8282
M. vote-16	0.8615	0.8565	0.8601	0.8546	0.8916	0.8875	0.8308	0.8240

## Data Availability

Not applicable.
